# The Potential of *In Vivo* Imaging for Optimization of Molecular and Cellular Anti-cancer Immunotherapies

**DOI:** 10.1007/s11307-018-1254-3

**Published:** 2018-07-20

**Authors:** Gilbert O. Fruhwirth, Manfred Kneilling, I. Jolanda M. de Vries, Bettina Weigelin, Mangala Srinivas, Erik H. J. G. Aarntzen

**Affiliations:** 1 Department of Imaging Chemistry and Biology, School of Biomedical Engineering and Imaging Sciences, Kings’ College London, London, UK; 2 Department of Preclinical Imaging and Radiopharmacy, Werner Siemens Imaging Center, Eberhard Karls University, Tuebingen, Germany; 3 Department of Dermatology, Eberhard Karls University, Tuebingen, Germany; 4 Department of Tumor Immunology, Radboud Institute for Molecular Life Sciences, Nijmegen, The Netherlands; 5 Genitourinary Medical Oncology and Koch Center, MD Anderson Cancer Center, Houston, USA; 6 Department of Cell Biology, Radboud Institute for Molecular Life Sciences, Nijmegen, The Netherlands; 7 Department of Radiology and Nuclear Medicine, Radboud University Medical Center, Geert Grooteplein 10, PO Box 9101, 6500 HB Nijmegen, The Netherlands

**Keywords:** Immunotherapy, *In vivo* imaging, Oncology, Translation, Imaging biomarker

## Abstract

This review aims to emphasize the potential of *in vivo* imaging to optimize current and upcoming anti-cancer immunotherapies: spanning from preclinical to clinical applications. Immunotherapies are an emerging class of treatments for a variety of diseases. The agents include molecular and cellular therapeutics, which aim to treat the disease through re-education of the host immune system, often *via* complex mechanisms of action. *In vivo* imaging has the potential to contribute in several different ways: (1) as a *drug development tool* to improve our understanding of their complex mechanisms of action, (2) as a *tool to predict efficacy*, for example, to stratify patients into probable responders and likely non-responders, and (3) as a *non-invasive treatment response biomarker* to guide efficient immunotherapy use and to recognize early signs of potential loss of efficacy or resistance in patients. Areas where *in vivo* imaging is already successfully implemented in onco-immunology research will be discussed and domains where its use offers great potential will be highlighted. The focus of this article is on anti-cancer immunotherapy as it currently is the most advanced immunotherapy area. However, the described concepts can also be paralleled in other immune-mediated disorders and for conditions requiring immunotherapeutic intervention. Importantly, we introduce a new study group within the European Society of Molecular Imaging with the goal to facilitate and enhance immunotherapy development through the use of *in vivo* imaging.

## Imaging as a Central Biomarker in Oncological Practice

Whole-body cross-sectional imaging played a seminal role in the era of chemotherapy and it remains the predominant technique for diagnosis and response monitoring [1]. Despite ongoing discussions about the accuracy of threshold values for quantitative imaging parameters (*e.g.*, largest tumor diameter, standard uptake values) in some patient subgroups [1–4] and the mediocre capacity to guide successful translation to clinical practice [5, 6], cross-sectional imaging and associated imaging biomarkers are fundamental to drug development in oncology. Key to the success of whole-body imaging was, first of all, its sensitivity to detect tumor lesions and assess disease stage, before clinical signs and symptoms become evident. Secondly, the fairly good correlation between disease burden as measured by anatomical imaging (biomarker) and the mechanism of action of chemo- and radiotherapy (decrease in tumor volume). Lastly, potential for standardization and its relative ease-of-use pushed anatomical imaging to become a core tool rendering various imaging biomarkers accessible [7, 8].

However, since the advent of immunotherapies, with their different and more complex mechanisms of action and thus different response dynamics, it became clear that the existing volume-based anatomical imaging biomarkers (*cf.* Response Evaluation Criteria in Solid Tumors (RECIST) criteria [1]) failed to adequately predict response. Consequently, adaptations to the original response criteria were explored. Immune-related RECIST (iRECIST) have been suggested as new response criteria tailored to immunotherapies [9, 10]. At this point in time, we can conclude that immunotherapies have changed the paradigm of anti-cancer treatment, but their mechanisms of action are much more diverse and complex than those of targeted therapies (epidermal growth factor receptor (EGFR) targeting mAbs, growth signal transduction protein kinase (*e.g.*, B-Raf) inhibitors *etc.*) or conventional therapies. Novel and accurate biomarkers continue to be essential to guide immunotherapy developments to secure early and optimal benefit for cancer patients. Whole-body *in vivo* imaging has great potential to significantly contribute in this context [7, 11–13], perhaps providing answers to some of the outstanding questions in the field of onco-immunology:

1. What immune cell types are involved and what cell types are critical for response?
2. How to assess the relation between target presence, density, affinity, and response?
3. What is the relevance of co-expressed features in the tumor microenvironment?
4. How to track *in vivo* distribution, fate, persistence, and function of cell therapies?
5. How to increase efficiency by rational design of combination therapies?
6. What are (early) spatial and temporal dynamics of response?
7. How different are responses between individual patients?

*In vivo* imaging has some unique features as compared to other biomarkers based on tissue/blood samples. In general, these advantages include (i) being non-invasive thereby overcoming issues associated with the tissue/blood sampling process, (ii) yielding whole-body information thereby overcoming under-sampling issues, (iii) providing kinetic information by dynamic imaging, and (iv) enabling standardization, which is particularly important for diagnosis and treatment monitoring across different hospitals/facilities. The *in vivo* imaging community currently faces the challenge to respond to the rapid developments in immunotherapy and how to optimally contribute with our tools and techniques.

## Current Issues in Anti-cancer Immunotherapy

When immunotherapy was adopted into oncological practice, it was met with great enthusiasm for two reasons: the durability of its responses and its efficacy in tumor types that were considered difficult to treat. Indeed, large randomized controlled trials on immune checkpoint inhibitors targeting cytotoxic T-lymphocyte-associated protein 4 (CTLA-4) or programmed cell death protein-1 (PD-1) and its ligand (PD-L1) pathways consistently demonstrated clinical efficacy in, *e.g.*, metastatic melanoma [14–16], non-small cell lung cancer [17–21], with typical response rates of 20–40 % that appeared to be durable [22]. Combined treatment with CTLA-4 and PD-1 mAbs increased the response rates in patients with advanced melanoma to > 50 % in line with enhanced severe adverse side effects [23]. However, there is also the sobering realization that besides combined therapies (CTLA-4 and PD-1 mAbs) in patients with metastatic melanoma, the majority of patients with other malignancies do not respond, experience serious side effects [24], or have tumors that are less amendable to treatment with immunotherapy. Tumor-infiltrating lymphocytes, low PD1/PD-L1 expression, and the presence of neo-antigens are likely associated with higher rates of response, but only provide a glimpse of the complex system that precludes response to immunotherapy [25].

Cellular anti-cancer immunotherapies have been proposed some time ago, involving redirecting immune cells to act on cells expressing tumor-associated antigens. But their development has been complex due to a variety of reasons (*e.g.*, live cell products, autologous *versus* allogeneic concepts, genetic engineering). Chimeric antigen receptor-based T cell therapies (CAR-T) are a personalized treatment that involves genetic engineering of patient-derived T cells to enable them to target the patient’s tumor cells. CAR-T generated great excitement [26] appearing to bring lasting control of cancers and even cancer cure within reach. Unprecedented clinical impact has been achieved employing CD19-targeted CAR-T in patients with refractory B cell malignancies with complete remissions in heavily pretreated patients [27–29]. Two products, tisagenlecleucel and axicabtagene ciloleucel, have recently gained FDA approval and both are CD19-targeted CAR-T products for the treatment of hematological cancers [30–32]. However, CAR-T also faces challenges. Most notably, results in solid tumors have so far been very disappointing, in part due to CAR-T not reaching their targets but also due to solid tumor microenvironments being severely immune suppressive. Moreover, many patients do not respond and exhibit resistance. Furthermore, patients may experience severe side effects including cytokine release syndrome (CRS), on-target off-tumor toxicity, and neurotoxicity [33, 34]. *In vivo* tracking of cell therapies by whole-body imaging has the potential to significantly aid the development of such cellular therapies. Due to the need for genetic engineering on the grounds of CAR introduction, there is now also the opportunity to co-encode additional payloads. That opened for the first time a convenient avenue for the introduction of non-immunogenic reporter genes into CAR constructs. These can be exploited for sensitive and repeated radionuclide imaging using short-lived radio isotopes, thereby rendering CAR-T *in vivo* tracking realistic.

Although molecular and cellular immunotherapies have transformed cancer treatment, the costs to patients and the healthcare system are staggering. Health technology assessments for FDA-approved molecular immunotherapies calculated approx. 80,000 euro per quality-adjusted-life-year [35–39]. To this, the costs of managing immune-related adverse effect should be added; approx. 20 % of patients experience major autoimmune effects [40], confirmed in post-marketing studies [41]. While monoclonal antibody-based treatments targeting immune-checkpoint inhibitors can be manufactured, distributed, and administered in ways resembling conventional processes, this is different for cellular immunotherapies. Both currently FDA-approved anti-CD19 CAR-T products require lympho-depletion prior to administration and are licensed for autologous use. This requires highly specialized and specific infrastructure/facilities (*e.g.*, on-site Good Manufacturing Practice (GMP) manufacturing of CAR-T products), high-level expertise (*e.g.*, specialized nursing staff and research teams), and is extremely cost-intensive (currently priced at almost US$500,000/year [30, 42]).

For immunotherapies to succeed in the long term, it will be paramount to optimize their efficacy in combination with efficient patient stratification and careful response monitoring to avoid unnecessary toxicities, application to the wrong patient groups, over-treatment, and keep cost affordable. Therefore, the following roles for *in vivo* imaging as a tool-assisting immunotherapy development and application processes should actively be investigated: (i) as a drug development tool to increase immunotherapy efficacy, (ii) as a predictive tool to ensure efficient immunotherapy application, and (iii) as an early response monitoring tool to reduce the risk of side effects.

## *In Vivo* Imaging as a Tool for the Development of Molecular and Cellular Immunotherapy

Improvements in the mechanistic understanding of the processes underlying cancers and their microenvironments including the associated interplay with the immune system will ultimately lead to the identification of new targets that can be exploited for anti-cancer treatments, be the basis for more efficacious combination therapies, and help overcome resistance phenomena [25, 43, 44]. In the preclinical setting, molecular imaging can be used to track the cancer cells or any immune cells of interest through cell tracking methodology. In the clinical setting, therapeutic cells may be rendered traceable *in vivo*, but any endogenous antigen-presenting cells (APC) including cancer cells rely on detection by conventional imaging approaches, as genome editing to express fluorescent proteins, receptors, or enzymes necessary for molecular imaging is not feasible in humans.

For *in vivo* cell tracking, it is necessary to label the cells with a contrast agent matching the desired imaging technology. There are two fundamentally different approaches to label cells, direct and so-called indirect labeling, both of which have been reviewed elsewhere [45]. Briefly, direct labeling means that the contrast agents are bound to or taken up normally *ex vivo* before administration to animals or humans. Indirect labeling means that the cells of interest express a reporter, either constitutively or induced by a certain event, which allows contrast generation *in vivo* upon administration of a suitable tracer. Direct cell labeling is fundamentally affected by label dilution and, in addition, label presence is not necessarily indicative of the initially labeled cell population. Consequently, indirect cell labeling is much better suited for long-term *in vivo* cell tracking, but it requires genetic engineering to implement the reporter genes. The latter need to be matched to the imaging modality of interest with manifold options available for highly sensitive cell tracking (using bioluminescence, fluorescence, and radionuclide technologies) [46–48], including endogenous reporters unlikely to cause any immunogenic response [49–51].

In the context of immunotherapies, all the above approaches and technologies have been reported including *ex vivo* radiolabeling [52], magnetic resonance imaging [53], nanoparticle-based imaging [54], and a variety of reporter gene methods [47, 51, 55, 56]. In preclinical rodent models, the migration pattern, local expansion, and retraction of transferred cell populations as well as systemic on-site off-target toxicities can be readily visualized during therapy and thereby guide optimization of current protocols. For example, adoptive T cell therapy can be enhanced when combined with co-immunostimulatory treatments, for instance, using a 4-1BB (CD137) agonistic antibody [57]. Similarly, adoptive Vγ9Vδ2 cell-based immunotherapy, which requires treatment with small molecule mevalonate pathway inhibitors [58], could be improved using imaging to optimize their uptake in solid tumors [59]. Intravital microscopy helped to elucidate how such co-treatments enhance both tumor infiltration and the cytotoxic activity of transferred T cells in mice [60]. Antigen-loaded dendritic cells (DCs) have been tested for stimulating tumor-antigen-specific immune responses in patients. Following transfer, DC migration and accurate positioning in lymph nodes is crucial for therapy success. Imaging allows tracking of the cells after transfer and showed that the route of administration is relevant for localization and function of transferred DCs [61–63]. Most notably, a recent study in glioma patients demonstrated proof-of-principle of applying multi-modal positron emission tomography (PET)-magnetic resonance imaging (MRI) to track reporter gene-expressing CAR-T in human brains to tumor sites with anatomical context afforded by MRI [64]. Furthermore, reporter gene technology can be configured to report inducible expression, which was exploited to image T cell activation in mouse models [65].

Multi-dimensional imaging allows simultaneous visualization of several molecular targets or cell populations and is an essential tool to investigate the synergistic effects of combination therapies. For example, bispecific antibodies can be used, which simultaneously bind surface markers on tumor cells and stimulate T cell co-receptors such as CD3. While tumor regression and enhanced T cell accumulation could be derived from alternative techniques, intravital microscopy uniquely allowed the monitoring of T cell dynamics in the tumor. It also revealed stable binding of multiple T cells to individual tumor cells as an underlying mechanism [66]. In search for effective combination therapies, tumor irradiation might modulate the tumor microenvironment to optimize immune checkpoint inhibition [67].

Likewise, imaging is a valuable tool to understand mechanisms, which cause failure of immunotherapy. For example, in immune checkpoint inhibition, little is known about interactions of checkpoint inhibitory antibodies with the host microenvironment within the tumor. Imaging allowed to track fluorescently labeled anti-PD-1 mAb in a mouse model of colon cancer and identified the removal of anti-PD-1 mAbs from its target T cells in a Fc receptor-dependent process by tumor-associated macrophages as unexpected resistance mechanism [68]. During anti-CD20 mAb therapy, Kupffer cells in the liver mediate B cell elimination by engulfing CD20-labeled B cells in a Fc-dependent manner [69]. These studies helped to optimize mAb design. In another example, the longitudinal tracking of chimeric antigen receptor (CAR) T cells labeled by [^18^F]NOTA-octreotide (NOTAOCT) in a mouse model demonstrated the superior targeting and expansion of micromolar affinity CARs for targeting intercellular adhesion molecule 1 (ICAM-1) overexpressing tumors which effectively improved efficacy and safety [70]. In the context of cell therapies, many clinical trials continue to be performed without knowledge about the location and fate of cells used in or derived from cell therapies *in vivo*, making it impossible to adequately monitor and assess safety. For example, in a recent clinical trial, patient deaths due to the immunotherapy exerting effects at the wrong physiological location [71] could have been prevented if the cells had been traceable and controllable. While suicide gene options for controllability have been developed [72, 73], imaging to inform about on-target off-site locations would be highly desirable to detect unsafe conditions, possibly early enough to avoid serious events.

Significant research activities have resulted in a variety of useful imaging tools that can be combined to allow simultaneous quantification of several parameters on the molecular, cellular, and organ levels, particularly in preclinical research but also becoming available for clinical research. In fact, the main limitations comparing preclinical with clinical settings in this context are not the available imaging tools, but rather the models itself, *i.e.*, questions regarding how adequately rodent models represent human disease.

## *In Vivo* Imaging as a Predictive Biomarker

For most immunotherapies, there is little solid understanding about the role of the presence and accessibility of their therapeutic target, which hampers efficient patient stratification. Clinical studies indicate a higher anti-PD-1 treatment efficacy in metastatic melanoma patients with strong PD-L1 expression at the tumor site when compared to patients with low expression [74]. Nevertheless, anti-PD-1 treatment is efficient even in patients without PD-L1 expression at the tumor site. As the PD-L1 expression status was suggested to correlate with response, radiolabeled antibodies and antibody fragments are being developed and first applications in patients are ongoing [75–77]. It is essential to reveal the temporal dynamics of PD-L1 expression, for example, during radiotherapy [78].

Whether non-invasive *in vivo* monitoring of the PD-L1 or PD-1 expression patterns is the most accurate target remains to be elucidated, regardless, it functions as a drug efficacy tool to assess target accessibility and target saturation. The same applies to other immune checkpoints that are currently under active investigation, such as CTLA-4, LAG-3, TIM-3, and OX40.

The current quest for accurate biomarkers to this purpose has already yielded an overwhelming number of publications on several domains of immunology [79]. It should therefore be pointed out that, at this period in time, imaging is suggested as a complementary tool to other methodologies, which can be more easily translated to routine clinical care with reasonable cost-benefit ratios.

## *In Vivo* Imaging to Monitor Treatment Response

Immunotherapy in general should result in increased numbers of effector immune cells to a critical level that is able to keep the immune response going [80]. Instead of following empirically designed treatment schedules, immunotherapy can probably be stopped as soon as it has achieved sufficient and persistent infiltration of immune cells in a patient. To the contrary, if immunotherapy fails to induce effective immune responses, patients should no longer be exposed to potentially hazardous immune-related adverse events and incremental costs to the healthcare system.

Non-invasive imaging of the activation status of the immune system might represent a promising tool to monitor the effects of immunotherapy. To this end, a [^89^Zr]desferrioxamine-labeled anti-CD8 and anti-CD4 cys-diabodies were developed and implemented for non-invasive *in vivo* PET tracking of endogenous CD8^+^/CD4^+^ T cells in mice and humans enabling the identification of immune checkpoint inhibitor therapy responders [81, 82]; a first clinical study with the anti-CD8 cys-diabody is currently ongoing. In line with this, a single domain CD8 mAb was established to monitor cancer immunotherapy. Homogeneous distribution of CD8^+^ T cell at the tumor site indicates response, whereas CD8^+^ T cell accumulation at the margins or lack of CD8^+^ T cell expression rather indicates lack of response [83]. Others have focused in preclinical studies with CD3-specific mAbs for detection of CD4^+^ and CD8^+^ T cells [84]. Another approach focuses on metabolic profiles during distinct phases of activation of immune cells, *e.g.*, monitoring deoxycytidine kinase activity with [^18^F]clofarabine ([^18^F]CFA); a first clinical study is currently running [85]. A similar approach is focused on quantifying enhanced glucose metabolism or cell proliferation of activated immune cells in the secondary lymphatic organs as a consequence of immunotherapy in humans [86]. Recently, imaging of OX40 expression, a T cell activation marker, has been preclinically implemented for immuno-PET ([^64^Cu]DOTA-AbOX40) representing promising novel tool for monitoring immunotherapies [87]. In addition, a granzyme-detecting radiotracer [88] as well as radio-labeled IL-2 targeting the IL-2 receptor enables detection of activated CD4^+^ and CD8^+^ T cells [89] with PET. Major histocompatibility complex (MHC) class II-specific mAbs were preclinically implemented for PET imaging of antigen-presenting cells (B cells, macrophages) and CD11b-specific radio-labeled mAbs for determination of myeloid cells such as macrophages and granulocytes [90]. Besides PET, MRI is another modality suitable for whole-body tracking *in vitro*-labeled adoptively transferred cells of interest or of *in vivo*-labeled endogenous phagocytic cells. Thus, endogenous monocytes and macrophages preferentially phagocytize systemic or locally injected emulsified perfluorocarbons (PFCs) and can be easily detected by ^19^F MRI [53, 91].

For better understanding of the basic underlining mechanisms of newly developed combined immunotherapies (agonistic and antagonistic immune checkpoint-specific mAbs or IDO inhibitors or chemotherapy or targeted therapies *etcetera*), it might be of paramount importance to track besides endogenous CD8^+^ and CD4^+^ T cells also regulatory CD4^+^ T cells, natural killer (NK) cells, B cells, neutrophils, macrophages, and dendritic cells at the tumor site as well as secondary and tertiary lymphoid organs. To this end, multimodal imaging approaches such as simultaneous PET/MRI measurements in the preclinical [92] and clinical settings [93] are of special importance to follow the fate of PFC-targeted monocytes/macrophages and radiolabeled mAb-targeted T cells simultaneously. Consequently, development of novel methods for *in vivo* targeting (labeling) cells of interest for multimodal imaging including optical imaging is needed.

## Outlook

**Fig. 1:**
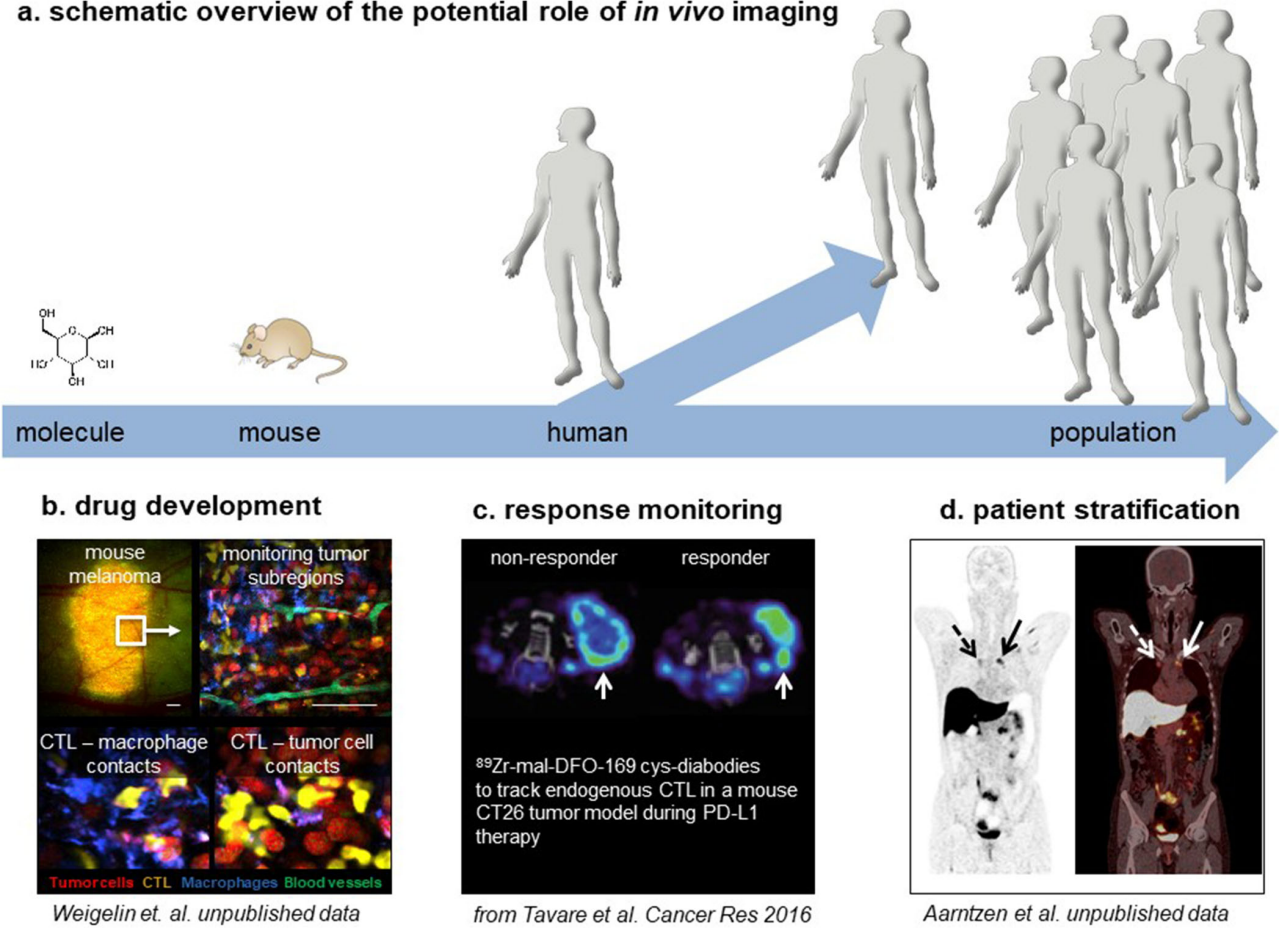
**a** Schematic overview of the different stages of onco-immunology research at which molecular imaging can potentially play a role. **b** Intravital multiphoton microscopy is applied to monitor immunotherapy response, *e.g.*, it allows to monitor adoptively transferred CD8+ T cell (CTL)(yellow) tumor (red) infiltration with high spatial resolution and to quantify interactions with other immune cells (macrophages, blue) or stromal elements (blood vessels, green) of the tumor microenvironment. **c** Preclinical PET imaging using ^89^Zr-mal-DFO-169 cys-diabodies to track endogenous CTLs in a mouse CT26 tumor model during PD-L1 therapy, demonstrating that response to PD-L1 inhibition coincides with infiltration of CTLs deep into the tumor (arrow, right panel), in contrast to non-responding mice in which CTL remain at the rim of tumors (arrow, left panel) [81]. **d** Coronal images of a [^18^F]fluoroestradiol PET/CT in a metastatic breast cancer patient, prior to start hormonal treatment, demonstrating estrogen-receptor expression in two mediastinal lymph node metastases (arrows) (of note, physiological high tracer uptake in the liver and excretion *via* the kidneys and urinary bladder)

During the development of immunotherapy, *in vivo* imaging has been indispensable by providing insights into the spatiotemporal dynamics of immune responses and the complex interactions between many different cell types at a molecular level. Currently, *in vivo* imaging is predominantly placed in the preclinical drug developmental phase of both cellular and molecular immunotherapy (Fig. 1). The full panel of molecular imaging techniques is exploited in mouse studies to identify targets or mechanisms of resistance and improve our understanding of the required elements for effective immunotherapy. Obvious areas of development area, the generation of new imaging tools for newly identified targets, and *in vivo* tracking of cellular therapeutics that do not rely on genetic engineering, hence, require labeling methodologies compatible with sensitive imaging but not interfering with cellular function. We consider the role of *in vivo* imaging established in this setting, offering unique tools to researchers in the field of onco-immunology.

Some techniques are currently reaching into clinical application, mostly in explorative studies that focus on early response monitoring. Imaging techniques that have successfully been translated, for example, are radiolabeling of therapeutic antibodies and *ex vivo* cell labeling, for clinical imaging with, *e.g.*, PET and MR. These techniques can be considered expensive and complex in terms of infrastructure, regulations, and logistics as compared to routine x-ray computed tomography (CT) or 2-deoxy-2-[^18^F]fluoro-D-glucose PET/CT imaging. However, they can also add important additional molecular information crucial to select the most effective treatments and apply them efficiently [7]. Importantly, mouse models are only partly representative of human disease, and this disparity will increase with the increasing complexity of combination therapies. Furthermore, patients can be highly individual in their disease and their response to treatment, and this individual monitoring is necessary. Early clinical validation is therefore necessary to increase attrition rates. Furthermore, the application of *in vivo* imaging for early response monitoring during immunotherapy can readily be cost-effective, when unnecessary treatments and toxicities are avoided. Notably, current immunotherapies have received approval based on clinical endpoints that were based on volume-based RECIST1.1 measured by CT or MRI, which are now demonstrated to underestimate the clinical benefit of patients, especially those with atypical response patterns.

For patient stratification, *in vivo* imaging might have a more difficult task to identify probable responders from a larger cohort of patients. For such application, *in vivo* imaging has competition from other potential biomarkers that provide off-the-shelf and high-throughput solutions like analyses of circulating tumor cells or tumor DNA, which for these reasons receive interest from the industry. We foresee that *in vivo* imaging will play a limited role in this domain, although implementation of *in vivo* imaging to support clinical decision making is being reported, for example, [^18^F]fluoroestradiol PET imaging in metastatic breast cancer [94, 95].

These considerations on the potential role of *in vivo* imaging can be extrapolated to other fields that increasingly apply cellular or molecular immunotherapy, *i.e.*, multiple sclerosis, diabetes type 1, transplantation and induction of tolerance [96, 97], neurodegenerative diseases [98], and infectious diseases [99]. Despite the promise of imaging the field of immunotherapy, several major hurdles remain. Successful application will require close collaborations between disparate groups, such as scientists, imaging specialists, contrast agent chemists, clinicians, and regulatory bodies.

## The New ESMI Onco-immunology and Therapy Study Group

The Onco-immunology and Therapy study group strongly believes that *in vivo* imaging is an essential part of the translation of new concepts and immunotherapies to the benefit of patients, by facilitating:

- Studies to further improve our mechanistic understanding of the immune system.
- Studies to better understand the mechanisms of action of existing and new immuno-therapies in preclinical and clinical settings.
- Hypothesis-driven design of new immunotherapies and combination therapies.

We therefore will reach out and advertise use of imaging in different immunotherapy areas to further spread implementation of imaging into onco-immunology and immunotherapy research through information and education. New scientific collaborations with related interest groups and scientific communities that foster the above-mentioned aims will be stimulated to identify needs and jointly seek for matching solutions.
